# Cardiac markers for risk stratification and prognosis in elderly patients with HFpEF

**DOI:** 10.3389/fmed.2026.1754295

**Published:** 2026-02-18

**Authors:** Omar Abusedera, Jana Sherif, Leena Karar, Dana Arekat

**Affiliations:** School of Medicine, Royal College of Surgeons in Ireland–Bahrain, Al-Muharraq, Bahrain

**Keywords:** biomarkers, BNP, elderly, galectin-3, HFpEF, NT-proBNP, prognosis, sST2

## Abstract

**Introduction:**

Heart failure with preserved ejection fraction (HFpEF) is increasingly prevalent among the elderly, particularly women, and is characterized by complex pathophysiology driven by comorbidities such as hypertension, diabetes, and renal dysfunction. These overlapping mechanisms make diagnosis and prognosis challenging, highlighting the importance of circulating biomarkers that reflect key biological processes such as myocardial stretch, fibrosis, inflammation, and injury.

**Objective:**

This mini-review explores the diagnostic and prognostic roles of major cardiac biomarkers BNP/NT-proBNP, high-sensitivity troponins, soluble suppression of tumorigenicity 2 (sST2), and Galectin-3 in elderly patients with HFpEF.

**Results:**

BNP and NT-proBNP remain the cornerstone biomarkers for diagnosing HFpEF, reflecting ventricular wall stress and providing strong prognostic value, although their interpretation in older adults can be influenced by renal function, age, and comorbidities. High-sensitivity troponins indicate chronic myocardial injury and have emerged as reliable predictors of mortality and recurrent hospitalizations. sST2 reflects inflammatory and fibrotic remodeling; while limited diagnostically, it shows consistent association with adverse outcomes. Galectin-3 captures ongoing myocardial fibrosis and structural remodeling, offering particularly strong prognostic information in HFpEF compared to HFrEF.

**Conclusion:**

Collectively, these biomarkers represent complementary windows into the pathophysiology of HFpEF. A multimarker strategy that integrates markers of wall stress, injury, inflammation, and fibrosis may enhance diagnostic precision and risk stratification in elderly patients, guiding more personalized approaches to management.

## 1 Introduction

Heart failure with preserved ejection fraction (HFpEF) is the most common type of heart failure in older patients, particularly women, and its prevalence rises steeply with age. The annual incidence of the disease doubles with each decade after age 65 for men and women (1). The pooled death rate for patients with preserved ejection is 121 per 1,000 patient-years according to a meta-analysis (2). Outcomes are particularly poor in hospitalized patients, where decompensation often leads to recurrent admissions or death.

HFpEF is usually driven by other comorbidities such as hypertension, diabetes, obesity, and chronic kidney disease. Chronic inflammation contributes to coronary microvascular dysfunction, and adverse cardiac remodeling through upregulation of E-selectins and intercellular adhesion molecule-1 expression, promoting cardiomyocyte hypertrophy and stiffness. The remaining clinical manifestations of HFpEF are typically the same as seen in other heart failure subtypes, including HFrEF (3). This complex biology makes single clinical parameters insufficient to capture the full spectrum of the disease mechanisms. These comorbidity-driven mechanisms not only shape HFpEF pathophysiology but are also reflected in distinct biomarker patterns, making biomarker assessment particularly valuable in elderly patients.

Biomarkers provide a crucial window into these pathophysiological processes. By reflecting myocardial stretch, injury, inflammation, fibrosis, and systemic dysfunction, circulating biomarkers offer the potential to improve diagnosis, refine risk stratification, and inform prognosis in elderly HFpEF. For example, Galectin-3 and soluble suppression of tumorigenicity 2 (sST2) show the extent of fibrosis and the severity of HFpEF (4). High-sensitivity troponins, which indicate ongoing myocardial injury and ischemia, have emerged as strong predictors of hospitalization and mortality in acute and chronic HFpEF (5). Although natriuretic peptides like B-type natriuretic peptide (BNP) and N-terminal proBNP (NT-proBNP) remain the gold standard biomarkers for diagnoses and prognosis in heart failure, their interpretation in elderly are limited by confounders such as renal dysfunction, obesity, atrial fibrillation, and age-related shifts in circulating concentrations (6).

Together, these biomarkers highlight the potential for a multimarker strategy to capture the heterogeneity of HFpEF in elderly patients. This mini-review will summarize current evidence on the prognostic and risk-stratification roles of BNP/NT-proBNP, high-sensitivity troponins, ST2, and galectin-3 in elderly HFpEF, with a focus on their clinical utility, limitations, and future implications for precision medicine.

## 2 BNP and NT-proBNP

### 2.1 Pathophysiology

Brain natriuretic peptide (BNP) and its N-terminal prohormone (NT-proBNP) are critical biomarkers in the assessment of heart failure with preserved ejection fraction (HFpEF). BNP is synthesized and secreted by the ventricles in response to ventricular stretching and pressure overload, signaling a compensatory mechanism to counteract heart failure (7). In contrast, NT-proBNP is an inactive fragment released during cleavage of proBNP and serves as a more stable marker in circulation, often exhibiting higher concentration levels than BNP itself (8). In elderly patients, the role of BNP is particularly significant due to its association with increased cardiovascular stress, fluid overload, and the body’s altered physiological response to aging (9). Elevated levels of BNP and NT-proBNP are indicative of worse outcomes and are essential for both diagnosis and risk stratification. These biomarkers not only reflect myocardial strain but also provide insights into the underlying pathophysiology of HFpEF, which often involves complex interactions between comorbidities such as atrial fibrillation, obesity, and renal impairment, all prevalent in older adults (10).

### 2.2 Diagnostic role in HFpEF

BNP and NT-proBNP remain the cornerstone biomarkers in diagnosing HFpEF. According to a 2021 systematic review and meta-analysis of 19 studies BNP demonstrated a pooled sensitivity of 78.7% (95% CI 71.9–84.2) and a specificity of 79.6% (95% CI 67.2–88.2) for diagnosing chronic HFpEF, performing comparably to or better than other emerging biomarkers. NT-proBNP showed marginally higher specificity but lower sensitivity. The AUC and In(DOR) of NT-proBNP and BNP were satisfactory for diagnostic purposes. Therefore, the reliability of NT-proBNP and BNP are equal as diagnostic markers for chronic HFpEF, given that both natriuretic peptides are in the same biological pathway. Due to the high specificity of NT-proBNP for HFpEF diagnosis, it is likely that NT-proBNP is more suitable for ruling out HFpEF. Higher sensitivity could be more applicable to secondary or tertiary care, whereas reliable specificity could be more important in primary care settings (11). Beyond diagnostic confirmation, natriuretic peptides and high-sensitivity cardiac troponins may serve as first-line screening biomarkers in patients with suspected HFpEF, particularly in elderly individuals with exertional dyspnea or equivocal imaging findings. Elevated biomarker levels can prompt a stepwise diagnostic strategy incorporating advanced echocardiographic techniques such as global longitudinal strain analysis to detect subclinical myocardial dysfunction. In selected cases, persistently abnormal biomarker profiles may support referral for cardiac magnetic resonance imaging to characterize myocardial fibrosis and adverse remodeling. Importantly, the clinical utility of BNP and NT-proBNP extends beyond single time-point risk assessment. Serial measurements provide dynamic insight into disease progression, ventricular remodeling, and treatment response, enabling longitudinal risk stratification. Evidence from heart failure with reduced ejection fraction populations demonstrates that reductions in natriuretic peptide levels correlate with improvements in functional capacity, quality of life, and clinical outcomes, including in real-world cohorts treated with sacubitril/valsartan (12). While extrapolation to HFpEF and valvular disease should be interpreted cautiously, these findings support the broader role of natriuretic peptides as longitudinal markers of myocardial stress and recovery in elderly patients.

### 2.3 Prognostic value and clinical application

Numerous studies have highlighted the prognostic value of BNP and NT-proBNP in elderly populations. Elevated levels correlate with increased rates of rehospitalization and mortality, making them essential tools for clinicians. For instance, in a prospective cohort of patients over 65 years presenting with elevated NT-proBNP, it was demonstrated that NT-proBNP levels were independent predictors of both short term (3 months) and long term (2 years) mortality, underscoring their prognostic value in elderly individuals with HfpEF (13). Moreover, the integration of BNP and NT-proBNP levels with clinical assessments enhances the ability to stratify risk and tailor management strategies more effectively in elderly with atypical symptoms and comorbidities (9). In elderly populations, interpretation of BNP and NT-proBNP requires careful consideration of age-related comorbidities that independently elevate natriuretic peptide concentrations. Chronic kidney disease, atrial fibrillation, obesity, and valvular heart disease—particularly aortic stenosis—are highly prevalent in patients over 65 years and may substantially influence circulating peptide levels. Recent evidence highlights the prognostic relevance of BNP and NT-proBNP in patients with severe aortic stenosis, where elevated concentrations predict symptom development and adverse cardiovascular outcomes independent of valve gradients. In the biomarker substudy of the EARLY TAVR trial, baseline NT-proBNP and high-sensitivity cardiac troponin T were associated with increased event rates in patients with asymptomatic severe aortic stenosis, supporting their role as markers of subclinical myocardial injury rather than valve obstruction alone (14). Similarly, the EVOLVED trial demonstrated that elevated NT-proBNP and hs-cTnT correlated closely with cardiac magnetic resonance evidence of myocardial fibrosis and adverse outcomes, reinforcing the concept that these biomarkers reflect maladaptive myocardial remodeling across elderly populations regardless of the primary disease trigger (15).

## 3 High-sensitivity troponin

### 3.1 Pathophysiology

Troponin elevation in heart failure can reflect myocardial injury in the absence of acute coronary syndromes, which suggests ischemia is not the only driver. Potential mechanisms include subendocardial ischemia, cardiomyocyte damage due to inflammatory cytokines, increased cell membrane permeability with leakage of cytosolic pool (16). Other studies have suggested that the increased preload seen in heart failure induces calcium entry which activates proteolytic enzymes such as calpains that degrade TnL and releases its fragments (17). Other potential drivers include elevated filling pressures, increased wall stress, endothelial dysfunction, tachycardia, arrhythmias, anemia, and hypotension (18). The exact mechanisms leading to TnL elevation in AHF are unknown, but multiple processes including both ischaemic and non-ischaemic mechanisms may contribute.

### 3.2 Diagnostic role in HFpEF

Troponin levels are typically higher in HFrEF than HFpEF. For HFpEF, elevated hs-cTnL at rest correlates with high pulmonary capillary wedge pressure, worsened systolic and diastolic function, and oxygen supply–demand mismatch, but not with myocardial oxygen demand (19, 20). Patients in the highest tertile of troponin levels have more than a two-fold risk of developing HFpEF compared to those in the lowest tertile, and chronic hs-cTnT elevation has been associated with adverse prognosis (19). Because elevated intracardiac pressure occurs in both types of heart failure, it is unclear whether the connection between filling pressures, reduced oxygen delivery, and troponin release is unique to HFpEF or a non-specific hemodynamic effect. Importantly, whether troponin elevation is merely a marker of adverse risk or reflects a mechanistic pathway of disease progression remains unresolved. Dynamic exercise studies show that hs-cTn rises disproportionately in HFpEF, tracking with worsening filling pressures and reduced cardiac reserve, which may enhance diagnostic sensitivity (21). Nevertheless, substantial variability in troponin elevations among HFpEF patients highlights the heterogeneity of the syndrome and limits its use as a standalone diagnostic tool.

### 3.3 Prognostic value and clinical application

Beyond its diagnostic implications, elevated troponin levels in HFpEF consistently predict adverse outcomes, including hospitalization, recurrent decompensation, and mortality. In a retrospective study enrolling 363 elderly patients with HFpEF decompensation, troponin levels ≥0.04 ng/mL were independently associated with higher 30-day, 1-year, and 2-year mortality, with a significant positive correlation between troponin and BNP (22). Similarly, another retrospective analysis found that hs-TnT ≥ 25.5 ng/L correlated with adverse cardiac events including all-cause mortality, non-fatal myocardial infarction, non-fatal stroke, and HF hospitalizations; patients with poor outcomes were older (23). Importantly, both studies demonstrated that cardiac troponin assays had a greater area under the curve than BNP, suggesting superior prognostic accuracy. These findings highlight the potential utility of troponin for risk stratification in elderly HFpEF populations, particularly when integrated into multimarker approaches.

## 4 sST2

### 4.1 Pathophysiology

sST2 is a promising biomarker for heart failure with preserved ejection fraction (HFpEF), reflecting underlying systemic inflammation, fibrosis, and cardiac remodeling. ST2 is upregulated in cardiac myocytes by mechanical strain or myocardial injury, however it can also be released from lungs or vascular endothelium signaling pulmonary congestion (24). Under normal conditions IL-33/ST2 signaling limits hypertrophy and fibrosis; however, elevated sST2 sequesters IL-33, blunting its cardioprotective effects and promoting adverse remodeling. In HFpEF, sST2 concentrations are associated with systemic inflammation, right ventricular dysfunction, and systemic congestion, but not with indexes of left ventricular (LV) morphology or function (25). Since sST2 reflects inflammation and fibrosis, while natriuretic peptides primarily reflect wall stress, the combination of the two may provide complementary biological insight and improve risk stratification.

### 4.2 Diagnostic role in HFpEF

So far, the evidence on sST2 being a diagnostic tool is limited. In a meta-analysis of five studies enrolling patients with a mean age of ~70 years, sST2 showed limited diagnostic accuracy for HFpEF compared to NT-proBNP, performing poorly in distinguishing HFpEF from healthy controls, hypertensive patients, and HFrEF (26). In another meta-analysis of three studies, sST2 demonstrates poor and inconsistent diagnostic performance for HFpEF (sensitivity 0.636; specificity 0.595), limiting its utility as a standalone biomarker (11). 13.5 ng/mL was found to be the best cutoff for predicting HFpEF onset (27). The predictive value of sST2 for HFpEF is worse than HFrEF, and is significant in basic and demographic-adjusted models including age but loses independent statistical significance when additional cardiovascular risk factors and robust cardiac biomarkers are included in the analysis, indicating that age adjustment attenuates but does not fully account for the influence of clinical confounders on sST2’s diagnostic utility (28). Again, this is explained by the lack of association with left ventricular function and structure. In comparison with the other markers, it has the least diagnostic role for HFpEF.

### 4.3 Prognostic value and clinical application

Although sST2 demonstrates limited utility as a diagnostic marker for HFpEF, particularly in distinguishing it from HFrEF or age-related comorbidities, its prognostic value is considerably more robust. In the same meta-analysis of men averaging 70 years old, higher serum sST2 was associated with increased all-cause mortality (multivariate HR 2.76, 95% CI 1.24–6.16) and the composite of all-cause death and HF hospitalization (multivariate HR 6.52, 95% CI 2.34–18.19) in HFpEF patients (24). Another prospective study of 70 year old men showed that In HFpEF patients, higher sST2 levels were independently associated with more adverse outcomes compared to HFrEF (HR 6.48 and HR 3.21, respectively) (29). This was similarly reported in a randomized controlled trial which demonstrated that sST2 levels correlated with worse outcomes in HFpEF compared to HFrEF (HR 10.04 and HR 3.28, respectively), even after adjusting for age, sex, and NYHA class. Mortality was also linked with sST2 levels (HR 12.39), however it was only a small number thus results were statistically insignificant (30). Overall, these findings highlight that while sST2 has limited diagnostic utility in HFpEF, it serves as a strong and independent prognostic biomarker, providing valuable insight for risk stratification and patient management.

## 5 Galectin-3

### 5.1 Pathophysiology

Galectin-3 plays a crucial role in the pathophysiology of cardiac fibrosis and remodeling. It is known to be produced by macrophages and monocytes, activating fibroblasts and causing fibrosis. Newer studies found that cardiomyocytes also release large amounts of Galectin-3 in response to mechanical stress (31). Activation of protein kinase C (PKC) by phorbol dibutyrate (PDBu) induces left ventricular hypertrophy along with increased galectin-3 expression and collagen production. When galectin-3 is inhibited (e.g., by *β*-lactose), collagen synthesis is blocked, suggesting that galectin-3 upregulation is a key step linking PKC activation to extracellular matrix remodeling in cardiac stress (32). Galectin-3 also contributes to the fibrotic process by promoting collagen synthesis and inhibiting collagen degradation, leading to myocardial stiffness and impaired function. Fibrosis is particularly relevant in elderly populations, where age-related changes in the myocardium predispose individuals to heart failure. The accumulation of extracellular matrix proteins, derived by Galectin-3, results in structural remodeling, which exacerbates heart failure symptoms (33).

### 5.2 Diagnostic role in HFpEF

Studies indicate that Galectin-3 has diagnostic value in HFpEF. For instance, an observational study found a significant correlation between circulating Galectin-3 concentrations and HFpEF, with markedly higher levels in affected patients compared to controls. The diagnostic accuracy of Galectin-3 was moderate (AUC 0.763, 95% CI 0.696–0.821, *p* < 0.01), with sensitivity and specificity of 76.0 and 71.9% at a threshold of 15.97 ng/mL, outperforming echocardiographic indices such as interventricular septal thickness and E/A ratio (34). Compared to NT-proBNP, Galectin-3 demonstrated superior diagnostic performance (AUC 0.71 vs. 0.59) and higher sensitivity and specificity (61 and 73% vs. 52 and 65%, respectively) (35). Furthermore, a 2021 meta-analysis reported that Galectin-3 exhibited high diagnostic potential (lnDOR = 2.94), comparable to the gold standard biomarkers NT-proBNP (lnDOR = 2.97) and BNP (lnDOR = 2.70). However, these findings were derived from only three studies, limiting the strength and generalizability of the results (11).

### 5.3 Prognostic value and clinical application

Early studies demonstrated that circulating galectin-3 levels are significantly elevated in patients with HFpEF, and higher baseline concentrations predict incident HF symptoms and adverse outcomes. This reflects its role as a mediator of myocardial fibrosis and ventricular remodeling, processes central to HFpEF progression. Its prognostic strength appears more pronounced in HFpEF than in HFrEF populations. In both acute and chronic HF settings, galectin-3 demonstrates predictive accuracy comparable to or exceeding NT-proBNP for short-term outcomes, including 60-day mortality (36). Furthermore, a meta-analysis reported that in HFpEF, elevated galectin-3 levels predicted all-cause death (HR: 1.55), composite endpoints (HR: 1.50), and cardiovascular death or hospitalization (HR: 1.71) (37).

## 6 Discussion

HFpEF remains a major clinical challenge due to its diagnostic uncertainty, biological heterogeneity, and lack of specific disease-modifying therapies. Elderly patients, in particular, present with overlapping comorbidities such as hypertension, diabetes, obesity, and renal dysfunction, which obscure distinguishing signs of HFpEF from normal aging or other chronic conditions (3). This multifactorial pathogenesis complicates not only diagnosis but also prognosis, as patients often exhibit heterogeneous pathophysiological mechanisms involving myocardial stiffness, inflammation, and systemic congestion. In this context, circulating biomarkers offer crucial mechanistic and clinical insights, bridging gaps in diagnostic precision, prognostic assessment, and potential therapeutic guidance.

Natriuretic peptides (BNP and NT-proBNP) remain the gold standard for HFpEF diagnosis due to their ability to reflect wall stress and left ventricular filling pressures (11). Their prognostic relevance is well established by predicting rehospitalization and mortality across age groups but interpretation in elderly patients is complicated by confounders like renal dysfunction, atrial fibrillation, and obesity. High-sensitivity cardiac troponins (hs-cTnT/I) serve as indices of chronic myocardial injury and microvascular ischemia. Persistent hs-cTn elevation in HFpEF cohorts often at concentrations below thresholds used for acute coronary syndromes has been consistently associated with increased mortality and hospitalization risk, surpassing BNP in discriminatory accuracy (22, 23). Troponin trends during exercise or decompensation phases further enhance diagnostic sensitivity for early HFpEF-related myocardial stress (21). Conversely, sST2 primarily reflects inflammatory and fibrotic remodeling. While its diagnostic accuracy for HFpEF is limited, high sST2 levels independently predict adverse outcomes, including cardiac death and heart failure hospitalization (11, 26). Lastly, galectin-3, a macrophage-derived profibrotic mediator, captures the chronic fibrotic and inflammatory milieu driving diastolic dysfunction. Its diagnostic accuracy is comparable with BNP and NT-proBNP and further studies with head-to-head comparison are needed (11, 35). Its prognostic influence appears stronger in HFpEF than in HFrEF, underscoring its role as a biomarker of myocardial stiffness and aging-related remodeling (36). A concise comparison of the diagnostic and prognostic roles of major cardiac biomarkers in elderly HFpEF is summarized in Table 1.

**Table 1:** Summary of major biomarkers in HFpEF: mechanistic domain, diagnostic accuracy, prognostic value and key limitations.

Biomarker	Primary pathophysiologic domain	Diagnostic utility	Prognostic value	Key limitations
BNP/NT-proBNP	Myocardial wall stress	Sensitivity 78–80%, specificity ~79%; cornerstone for HFpEF diagnosis	Predicts hospitalization and mortality	Influenced by age, renal dysfunction, AF, obesity
hs-Troponin I/T	Myocardial injury/microischemia	Moderate diagnostic role; correlates with PCWP and diastolic dysfunction	Strong predictor of mortality, recurrent decompensation	Non-specific elevation in renal disease, sepsis
sST2	Inflammation and fibrosis (IL-33/ST2 pathway)	Poor diagnostic accuracy (AUC ~ 0.64)	Strong independent predictor of death and HF hospitalization	Confounded by age, systemic inflammation
Galectin-3	Fibrosis and remodeling	Moderate diagnostic accuracy (AUC ~ 0.76)	Strong predictor of CV death, hospitalization; stronger in HFpEF than HFrEF	Elevated in renal and hepatic fibrosis; limited by small studies

**Figure 1:**
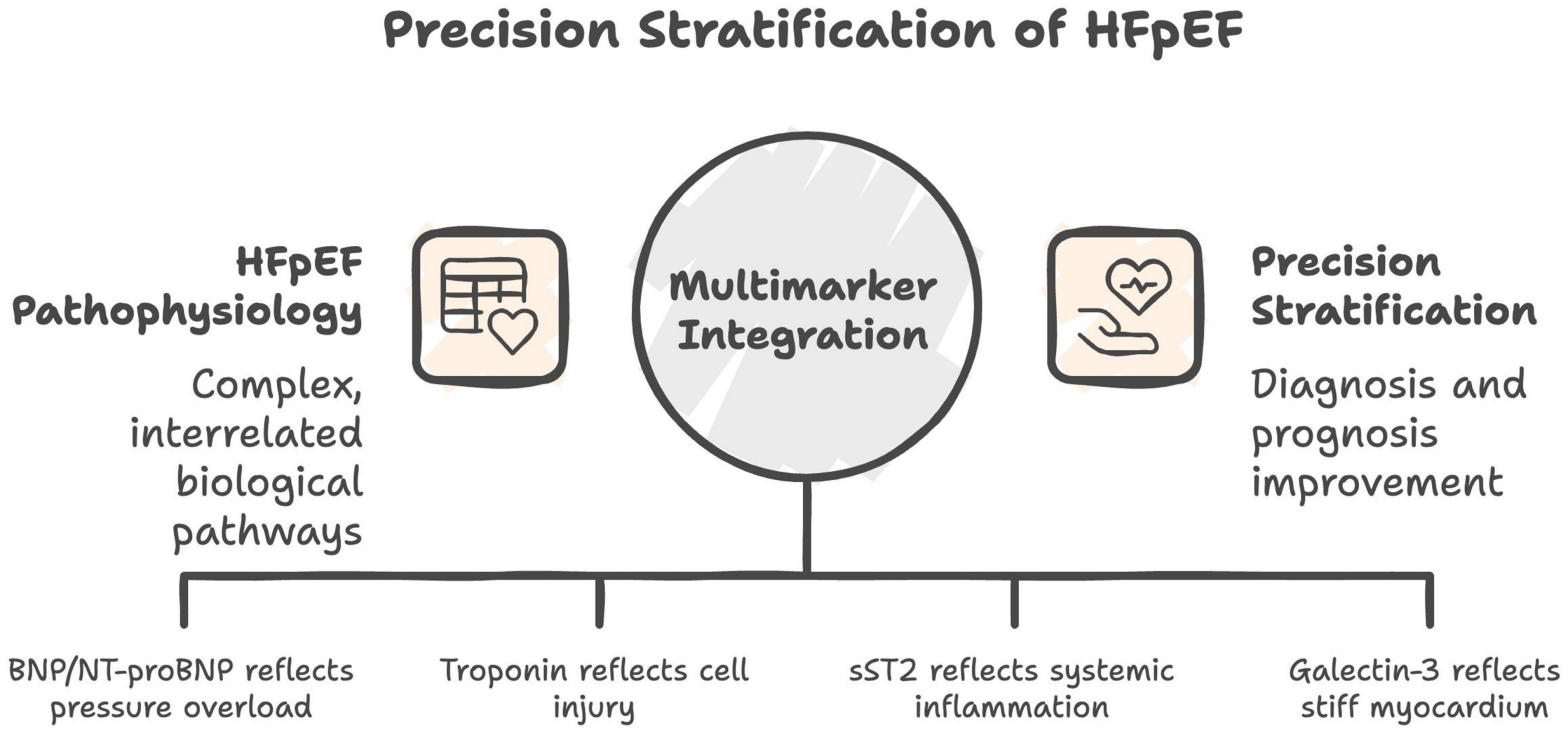
Conceptual model illustrating the role of multimarker integration in the precision stratification of HFpEF.

As shown in Table 1, each biomarker provides complementary mechanistic and prognostic insight, underscoring the potential value of an integrated multimarker strategy for more precise risk stratification (see Figure 1). However, several limitations temper clinical utility: cutoff values vary widely across studies; confounding by age, obesity, and renal impairment remains unresolved; and inconsistent HFpEF definitions impede generalizability. Moreover, no single biomarker achieves sufficient sensitivity and specificity for diagnostic confirmation in isolation, encouraging combining biomarkers with imaging (e.g., strain analysis) and hemodynamic testing. The limited number of studies along with potential bias within these studies should be accounted for.

Future research should focus on integrated diagnostic algorithms and phenotype-specific biomarker panels that combine clinical, echocardiographic, and molecular data to enhance diagnostic precision. Machine-learning and omics approaches could unravel novel biomarker patterns representing distinct HFpEF endotypes, aiding both risk stratification and therapeutic targeting. Ultimately, large-scale validation in diverse elderly populations remains essential to translate biomarker-guided strategies into clinically actionable frameworks for a disease that continues to lack effective targeted treatments.

## 7 Conclusion

In conclusion, the use of circulating biomarkers in elderly HFpEF provides meaningful advances in diagnosis, prognosis, and risk stratification, adapting to the syndrome’s complex and multifactorial nature. Traditional markers such as BNP and NT-proBNP remain crucial for initial assessment, but their diagnostic accuracy is diminished by age-related confounders and comorbidities. Newer biomarkers including high-sensitivity troponins, sST2, and galectin-3 offer mechanistic and prognostic information, particularly reflecting injury, inflammation, and fibrosis, and galectin-3 especially shows stronger prognostic value in HFpEF than HFrEF. However, variability in cutoff values, study design, and patient populations limits the reliability of any single test. Integrative, multimarker strategies combined with clinical and imaging data hold the most promise for improving precision in diagnosis and personalization of therapy, though ongoing validation in large, diverse cohorts is essential before widespread adoption.
